# Imaging-genetics of sex differences in ASD: distinct effects of *OXTR* variants on brain connectivity

**DOI:** 10.1038/s41398-020-0750-9

**Published:** 2020-03-03

**Authors:** Leanna M. Hernandez, Katherine E. Lawrence, N. Tanya Padgaonkar, Marisa Inada, Jackson N. Hoekstra, Jennifer K. Lowe, Jeffrey Eilbott, Allison Jack, Elizabeth Aylward, Nadine Gaab, John D. Van Horn, Raphael A. Bernier, James C. McPartland, Sara J. Webb, Kevin A. Pelphrey, Shulamite A. Green, Daniel H. Geschwind, Susan Y. Bookheimer, Mirella Dapretto, On behalf of the GENDAAR Consortium

**Affiliations:** 1 Ahmanson-Lovelace Brain Mapping Center, University of California, Los Angeles, Los Angeles, CA 90095 USA; 2 Department of Psychiatry and Biobehavioral Sciences, University of California, Los Angeles, Los Angeles, CA 90095 USA; 3 Department of Neurology, David Geffen School of Medicine, University of California, Los Angeles, Los Angeles, CA 90095 USA; 4 Autism & Neurodevelopmental Disorders Institute, The George Washington University, Washington, DC USA; 5 Center for Integrative Brain Research, Seattle Children’s Research Institute, Seattle, WA USA; 6 Department of Pediatrics, Harvard Medical School, Boston, MA USA; 7 USC Mark and Mary Stevens Neuroimaging and Informatics Institute, Laboratory of Neuro Imaging, Keck School of Medicine of USC, University of Southern California, Los Angeles, CA USA; 8 Department of Psychiatry and Behavioral Sciences, University of Washington, Seattle, WA USA; 9 Department of Pediatrics, Yale School of Medicine, New Haven, CT USA; 10 University of Virginia School of Medicine, Charlottesville, VA USA; 11 Department of Human Genetics, University of California, Los Angeles, Los Angeles, CA 90095 USA

**Keywords:** Autism spectrum disorders, Clinical genetics

## Abstract

Autism spectrum disorder (ASD) is more prevalent in males than in females, but the neurobiological mechanisms that give rise to this sex-bias are poorly understood. The female protective hypothesis suggests that the manifestation of ASD in females requires higher cumulative genetic and environmental risk relative to males. Here, we test this hypothesis by assessing the additive impact of several ASD-associated *OXTR* variants on reward network resting-state functional connectivity in males and females with and without ASD, and explore how genotype, sex, and diagnosis relate to heterogeneity in neuroendophenotypes. Females with ASD who carried a greater number of ASD-associated risk alleles in the *OXTR* gene showed greater functional connectivity between the nucleus accumbens (NAcc; hub of the reward network) and subcortical brain areas important for motor learning. Relative to males with ASD, females with ASD and higher *OXTR* risk-allele-dosage showed increased connectivity between the NAcc, subcortical regions, and prefrontal brain areas involved in mentalizing. This increased connectivity between NAcc and prefrontal cortex mirrored the relationship between genetic risk and brain connectivity observed in neurotypical males showing that, under increased *OXTR* genetic risk load, females with ASD and neurotypical males displayed increased connectivity between reward-related brain regions and prefrontal cortex. These results indicate that females with ASD differentially modulate the effects of increased genetic risk on brain connectivity relative to males with ASD, providing new insights into the neurobiological mechanisms through which the female protective effect may manifest.

## Introduction

Across species, the neuropeptide oxytocin plays a critical role in a wide range of reproductive and complex social processes, including initiation of uterine contractions during childbirth, lactation, pair bonding, and social reward processing^1,2^. The effects of oxytocin are dependent on expression of its receptor throughout the brain and body, and several variants in the oxytocin receptor gene (*OXTR*) have been linked to increased rates of autism spectrum disorder (ASD)^3–6^. As social deficits are a core feature of ASD, the oxytocin system has attracted considerable attention as a potential target for pharmacological manipulation in individuals with autism^7–9^. Indeed, in males with ASD, treatment with intranasal oxytocin has been shown to improve social responsivity, seemingly through effects on reward-related subcortical brain areas and frontal brain regions important for social cognition^10–16^.

Given that males are about four times as likely to receive an ASD diagnosis as females^17^, it is not surprising that much of the research conducted to date on the neurobiological correlates of oxytocin functioning in individuals with ASD has been in males. However, sex-specific regulation of the oxytocin system has been reported in both animals^18,19^ and humans^20^, suggesting that findings in males may not be generalizable to females. Importantly, animal studies have shown sex-differences in *OXTR* expression in several brain regions important for social functioning and maternal behavior, with females displaying higher expression relative to males^21,22^, and that genetic variation in the *OXTR* affects the expression of receptors in subcortical brain areas important for social behavior^23^. The neurobiological effects of oxytocin suggest that it may have a protective effect by enhancing neural plasticity through induction of long term potentiation^24–26^ at synapses where the *OXTR* is expressed^27,28^, and by reducing neuroinflammation through inhibition of microglial activation^27,29^. This evidence for a role of oxytocin in modulating neuroplasticity and inflammation, as well as evidence for brain-based sex-differences in functioning of the oxytocin system, has led some to hypothesize that oxytocin may have a neuroprotective effect against the development of ASD in females^30^.

In the genetics literature, theories on the neurogenetic underpinnings of sex differences in ASD have hypothesized a female protective effect (FPE) whereby females require a higher burden of genetic and environmental risk factors to develop ASD^31^. Evidence for a FPE comes from research showing that females with ASD carry more deleterious copy number and single nucleotide variants relative to males with ASD^32,33^, and further that genes highly expressed in the male brain also tend to be upregulated in the brains of individuals with ASD^34^. Together, these studies suggest that the male-bias in ASD may be due to inherent differences in sex-specific neurobiology, which in turn affect the impact that ASD risk variants have on neuroendophenotypes^34,35^. At the neural systems level, sex differences in the capacity of the brain to adapt to genetic and environmental risk factors may mediate the effects of genetic risk for ASD on the brain and ultimately behavior^36^; indeed, genes involved in the regulation of synaptic plasticity have been associated with increased ASD risk^37^.

As multiple SNPs in the *OXTR* have been linked to higher rates of ASD^3–6^ and altered neural activity to rewards^38^, the oxytocin system serves as an ideal model in which to test whether increased genetic risk for ASD affects neural functioning in a dose-dependent manner. In the brain, the oxytocin receptor is expressed in subcortical areas, with enhanced expression in reward-related areas including the NAcc, hypothalamus, and substantia niagra^39^— regions which also show altered function and connectivity in individuals with ASD^40–44^. A recent imaging-genetics study of predominantly male youth with and without ASD showed that in the presence of increased genetic risk for ASD in the *OXTR*, youth with ASD display reduced connectivity between the NAcc and other subcortical brain regions critical for implicit learning and reward processing^45^. Conversely, typically developing youth with a greater number of ASD-associated variants in the *OXTR* gene display compensatory functional connectivity between the NAcc and prefrontal cortex, which may buffer them from expressing social cognitive deficits indicative of ASD^45^. However, researchers have yet to assess potential sex-differences in the dose-dependent relationship between genetic risk for ASD and neuroendophenotypes, and to investigate putative sex-dependent mechanisms of neural plasticity and resilience. Using data from a multisite study focused on gender differences in ASD, here we examined how ASD risk-allele-dosage in the *OXTR* relates to heterogeneity in NAcc network connectivity and, critically, investigate sex differences in the effects of genetic risk on the brain by comparing cohorts of males and females with and without ASD.

## Methods

### Subjects

Female participants were high functioning females with ASD (*n* = 50) and neurotypical (NT) females (*n* = 52) between the ages of 8 and 17 (*M* = 13.66, *SD* = 2.67) who were recruited from four sites (Harvard, Seattle Children’s Hospital, University of California, Los Angeles (UCLA), and Yale) as part of the Gender Exploration of Neurogenetics and Development to Advance Autism Research (GENDAAR) multisite consortium. Male participants were high-functioning males with ASD (*n* = 37) and NT males (*n* = 34) between the ages of 8 and 17 (*M* = 13.34, *SD* = 2.05) who were recruited from the Los Angeles area through the UCLA Center for Autism Research and Treatment (CART). Note that male subjects in the current study are the same as described in Hernandez et al.^45^; the sample in that study included several female participants (four ASD, seven NT), which were excluded in the current study where analyses were re-run in NT and ASD male-only subsamples. All previously reported results held (Supplementary Figs. 1, 2). The data is publicly available through the NIMH Data Archive (Collection ID 10 and 2021).

Participants with a history of claustrophobia, diagnosed neurological disorders, genetic conditions, structural brain abnormalities, or metal implants were excluded from study participation. Study protocols were approved by the Institutional Review Board at each participating site and informed consent and assent to participate in research were obtained from legal guardians and study participants. All participants with ASD had a prior diagnosis for the disorder based on the diagnostic and statistical manual of mental disorders^46^, which was confirmed using the Autism Diagnostic Observation Schedule—2nd Edition (ADOS-2)^47^, Autism Diagnostic Interview-Revised (ADI-R)^48^, and best clinical judgment by licensed clinicians at each participating site. Criterion for study inclusion was an IQ above 70 as assessed by the Differential Ability Scales II^49^, Wechsler Intelligence Scale for Children-IV^50^, or the Wechsler Abbreviated Scale of Intelligence^51^. Additional behavioral measures included the Social Responsiveness Scale (SRS)^52^, a measure that provides a quantitative index of autistic traits. The SRS is designed to assess reciprocal social behavior in both NT and ASD individuals ages 4–18 years; questionnaires were completed by parents of study participants. Seventeen ASD females and 18 ASD males reported use of one or more psychotropic medications (Supplementary Table 1). A summary of demographic information is presented in Table 1.

**Table 1:** Sample descriptives.

	ASD Females	ASD Males	NT Females	NT Males	Females ASD vs. NT	Males ASD vs. NT	ASD Female vs. Male	NT Female vs. Male
Age	13.84	13.50	13.49	13.17	0.59	0.50	0.53	0.60
Full IQ	97.91	105.24	112.97	106.26	0.001^b^	0.72	0.08	0.03^a^
Nonverbal IQ	99.53	105.65	110.82	105.32	0.009^b^	0.92	0.12	0.11
Verbal IQ	101.16	103.73	111.18	107.09	0.018^a^	0.23	0.50	0.19
ADOS social	7.97	9.70	–	–	–	–	0.05^a^	–
ADOS repetitive behavior	2.06	2.29	–	–	–	–	0.57	–
ADOS severity score	6.13	7.02	–	–	–	–	0.10	–
ADI social	19.09	20.11	–	–	–	–	0.41	–
ADI Communication	15.77	16.30	–	–	–	–	0.64	–
ADI repetitive behavior	6.00	6.78	–	–	–	–	0.21	–
Mean relative motion	0.12	0.08	0.09	0.08	0.16	0.27	0.02^a^	0.10
Mean volumes scrubbed	14.97	2.81	8.58	2.97	0.21	0.89	0.003^b^	0.07
Mean *OXTR* risk variants	3.44	3.30	3.45	3.12	0.94	0.47	0.58	0.19
Subjects with ≥1 risk allele								
rs1042778	28	30	27	23	0.53	0.19	0.47	0.18
rs2254298	6	7	8	9	0.59	0.45	0.99	0.83
rs53576	21	20	21	12	0.87	0.11	0.33	0.02^a^
rs237887	28	27	25	29	0.22	0.20	0.14	0.32
Self-reported ethnicity								
Asian	2	2	3	3	0.67	0.57	0.88	0.97
Black/African American	0	2	3	6	0.08	0.10	0.18	0.31
Other/Mixed	4	4	5	2	0.76	0.46	0.83	0.22
White	26	25	22	19	0.18	0.31	0.20	0.37

Mean values and *p*-values derived from two-tailed independent samples *t*-tests or chi-squared tests. ^a^*p* < 0.05; ^b^*p* < 0.01.

*ASD* autism spectrum disorder, *NT* neurotypical, *IQ* intelligence quotient, *ADOS* Autism diagnostic observation schedule, *ADI* Autism Diagnostic interview, *OXTR* oxytocin receptor gene.

### Genotyping

Genomic DNA from whole blood was obtained from the Rutgers University Cell and Data Repository (RUCDR) or extracted using standard protocols (Gentra Puregene Blood DNA extraction kit; Qiagen). Genotyping for SNPs rs237887 and rs1042778 was performed at Yale University or the UCLA Neuroscience Genomics Core (https://www.semel.ucla.edu/ungc) according to standard manufacturer protocols using the HumanOmni2.5-8 BeadChip microarray (Illumina Inc.) Commercially available TaqMan assays were used to genotype SNPs rs53576 and rs2254298 at the UCLA Genotyping and Sequencing Core (GenoSeq Core; http://genoseq.ucla.edu) using a 5′ nuclease assay to discriminate between the two alleles (Taqman SNP Genotyping Assay, Applied Biosystems Inc.). Polymerase chain reactions were performed using 5-μL reaction volumes in 384-well plates with 25 ng of DNA and Taqman genotyping master mix from Applied Biosystems Inc. End point reads of fluorescence levels were obtained with a Roche 480 lightcycler following manufacturer’s protocol. After quality filtering (<5% missing per person/per SNP, >1% minor allele frequency, Hardy-Weinberg equilibrium *p* > 10^−7^), multi-dimensional scaling was performed in PLINK (http://pngu.mgh.harvard.edu/purcell/plink/) using the default settings with the HapMap 3 reference panel (http://hapmap.ncbi.nlm.nih.gov/).

### MRI data acquisition

Resting-state functional magnetic resonance imaging (rs-fcMRI) data were collected at each of the four GENDAAR participating sites on either a Siemens 3T Trio (12-channel head coil) or a Prisma 3T (20-channel head coil) whole-body scanner. For each subject, a T2*-weighted resting-state functional MRI sequence was acquired (6–8 min, TR = 2000ms, TE = 30 ms, FOV = 192 mm, 34 slices, slice thickness 4 mm, in-plane voxel size 3 × 3 mm for females; 6 min, TR = 3000 ms, TE = 28 ms, FOV = 192 mm, 34 slices, slice thickness 4 mm, in-plane voxel size 3 × 3 mm for males). For registration purposes, a matched-bandwidth echo-planar scan was also acquired (Siemens Trio: TR = 5000 ms, TE = 34 ms, FOV = 192 mm, 34 slices, slice thickness 4 mm, in-plane voxel size 1.5 × 1.5 mm; Siemens Prisma: identical parameters except for TE = 35 ms, 36 slices).

### fMRI data analysis

The rs-fMRI data were analyzed using FSL (FMRIB’s Software Library, www.fmri.ox.ac.uk/fsl)^53^ and AFNI (Analysis of Functional NeuroImages)^54^. Data were processed using the same pipeline described in Hernandez et al.^45^ to ensure valid comparisons of results obtained for the male and female groups. Images were skull-stripped using AFNI. Next, FSL’s Motion Correction Linear Registration Tool (MCFLIRT)^55^ was used to align the functional images with the mean functional volume. Translations in the *x*, *y*, and *z* dimensions were calculated from volume to volume, then averaged to create a measure of mean displacement. Functional data were linearly registered to the matched-bandwidth EPI volume (6 degrees of freedom), followed by registration to the MNI 152 2 mm standard brain (12 degrees of freedom). FSL’s Automatic Segmentation Tool (FAST) was applied to high-resolution scans to create masks for grey matter, white matter, and cerebral spinal fluid for each subject; these variables, as well as the global signal were regressed from the data using FSL’s FEAT. Images were smoothed using a 5 mm FWHM Gaussian kernel. Bandpass filtering was applied to the data (0.1 Hz > *t* > 0.01 Hz).

Motion scrubbing was performed, including removal of volumes for which frame-wise displacement exceeded 0.5 mm and DVARS exceeded 0.5%; two volumes preceding and one volume following a scrubbed image were also removed^56^. Participants with <5 min of data after scrubbing were excluded from final analyses resulting in a final sample of 32 ASD females, 33 NT females, 37 ASD males, and 34 NT males. The mean number of volumes remaining after scrubbing were 176, 179, 117, and 117, for ASD females, NT females, ASD males, and NT males, respectively. There were no differences in the number of volumes remaining between ASD and NT females, or between ASD and NT males. There were significant differences in the mean number of volumes remaining between female and male participants (*p* < 0.01), thus, the number of remaining volumes after scrubbing for each participant was used as a covariate in group-level analyses comparing male and female youth. FSL’s FLIRT was used to register residuals from the motion scrubbed data to the EPI volume (6 degrees of freedom), followed by registration to the MNI 152 2 mm standard brain (12 degrees of freedom). Power calculations were conducted in G*Power 3.1;^57^ anticipated effect size estimates were obtained from previously published work assessing the effect of *OXTR* variants on reward network activity^38^. Sample sizes for males and females with and without ASD in the current study are sufficient to detect significant effects in each group at 80% power and a 0.05 significance level.

Statistical analyses were performed using the general linear model in FSL’s FEAT. The NAcc was defined bilaterally using the Harvard Oxford Atlas at a probability threshold of 25%. Average timeseries were extracted from the bilateral NAcc and correlated with every other voxel in the brain to generate functional connectivity maps for each participant. Individual subject-level correlation maps were transformed to *z*-statistic maps using Fisher’s *r* to *z* transform. Single-subject maps were combined in higher-level group analyses in FSL’s FEAT using FLAME 1 + 2, a mixed effects model. Female diagnostic groups (i.e., female ASD vs. NT) did not differ in age; however, as diagnostic groups differed in IQ and the number of participants who underwent MRI at each site (Table 1) these variables were used as covariates in group-level comparisons. Male diagnostic groups (i.e., male ASD vs. NT) did not show any between-group differences in demographic variables; accordingly, comparisons between male diagnostic groups did not include covariates. Results of NAcc whole-brain connectivity analyses are presented at z > 3.1 (*p* < 0.001), corrected for multiple comparisons at *p* < 0.05. Group-level analyses testing the interaction between sex and diagnostic status on NAcc connectivity were controlled for demographic variables on which male and female groups differed including MRI data collection site, IQ, and number of functional volumes remaining after motion scrubbing. Examination of sex differences in positive and negative NAcc connectivity focused on regions showing significant connectivity (*z* > 3.1, cluster-corrected at *p* < 0.05) in any group (NT females, NT males, ASD females, ASD males).

To assess the aggregate effect of *OXTR* genetic risk on NAcc connectivity, cumulative genetic risk scores were computed for each participant as the sum of the number of ASD-associated risk alleles across four *OXTR* SNPs: rs53576, rs237887, rs1042778, rs2254298 (Supplementary Table 2). Mean genetic risk was calculated across NT and ASD groups; subjects’ demeaned genetic risk score was entered as a regressor in higher-level FEAT analyses (FLAME 1 + 2). We previously demonstrated that none of the four SNPs contributed disproportionally to altered NAcc-resting state functional connectivity by iteratively assessing effects of three SNPs on brain connectivity in a leave-one-out fashion; results when assessing 3-SNP models were identical to the final 4-SNP model^45^. Modulation of NAcc connectivity by aggregate *OXTR* risk was assessed at *z* > 3.1, corrected for multiple comparisons at *p* < 0.05.

## Results

Male results for both whole-brain NAcc connectivity and effects of *OXTR* risk variants mirrored those previously reported in a largely overlapping sample of ASD and NT participants^45^. Greater numbers of ASD-associated variants in the *OXTR* gene were associated with reduced within-network connectivity in males with ASD, and greater connectivity between the NAcc and prefrontal cortex in NT males (Supplementary Figs. 1, 2). Below, we first detail the results for *females* with and without ASD, and then examine the interaction between sex, diagnosis, and genetic risk for ASD in the *OXTR*.

### *OXTR* genetic variation in females

In females, the average number of risk alleles in the *OXTR* was 3.45 (*SD* = 1.03, range 2–6); NT females had on average 3.45 *OXTR* risk alleles (*SD* = 1.03), ASD females had on average 3.44 *OXTR* risk alleles (*SD* = 1.05; Table 1). An independent samples t-test indicated that, as expected, there was not a significant difference in the average number of *OXTR* risk alleles in NT and ASD females. As previously reported in ASD males^45^, inheriting greater numbers of *OXTR* risk alleles was associated with higher calibrated severity scores on the ADOS in females with ASD (*r* = 0.29, one-tailed *p* = 0.05). There were no differences between males and females with and without ASD in the cumulative number of inherited *OXTR* risk-alleles (Table 1), nor any relationships between IQ or medication status and the number of ASD-associated *OXTR* risk alleles.

### Female NAcc whole-brain connectivity

**Fig. 1:**
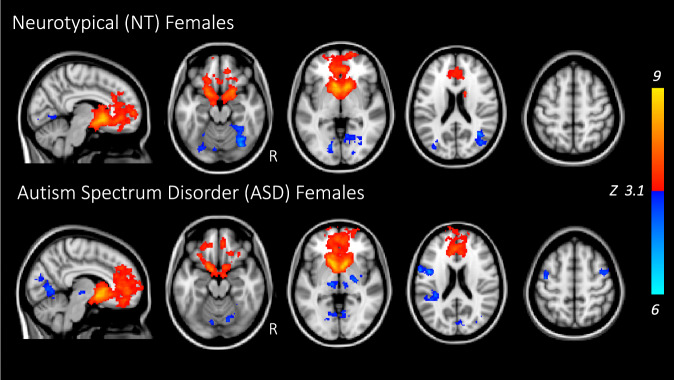
Nucleus accumbens (NAcc) whole-brain connectivity in females with and without ASD. Red/yellow indicate positive connectivity with the seed; blue/cyan indicate negative connectivity with the NAcc seed. Maps are shown at *z* > 3.1, corrected for multiple comparisons at *p* < 0.05. NT neurotypical; ASD autism spectrum disorder.

The NAcc showed significant connectivity with other key reward-related brain regions in both NT females and females with ASD (Fig. 1). Both groups showed positive connectivity between the NAcc and frontal pole, superior frontal gyrus, frontal medial and orbital cortex, paracingulate and cingulate cortex, caudate, and putamen; connectivity between NAcc and amygdala was observed in NT females only. Negative (anticorrelated) connectivity was observed between the NAcc and occipital cortex (lingual and fusiform gyri). Females with ASD displayed additional negative connectivity between the NAcc and sensorimotor brain regions (bilateral middle frontal gyrus and thalamus, left precentral gyrus, right pallidum and insula) and areas involved in auditory/language processing (i.e., middle temporal gyrus, angular gyrus, parietal operculum). These results lend support to findings previously reported in NT and ASD males, who showed positive connectivity between the NAcc and frontal cortex, anterior cingulate, bilateral caudate and putamen (with NT males having additional NAcc-precuneus positive connectivity), and negative connectivity to thalamus, occipital, and parietal cortex (Supplementary Figs. 1, 2)^45^. There were no significant differences between NT females and ASD females in NAcc whole-brain positive or negative connectivity.

Next, we compared whole-brain NAcc connectivity between males and females with and without ASD. Sex differences were observed in ASD youth; relative to ASD males, ASD females showed stronger negative connectivity between the NAcc and right lingual gyrus (MNI coordinates: 14, −64, −4; 105 voxels; max *z* = 4.33). No sex differences were observed when comparing NT males and females.

### Cumulative genetic risk: effects on NAcc connectivity in females

**Fig. 2:**
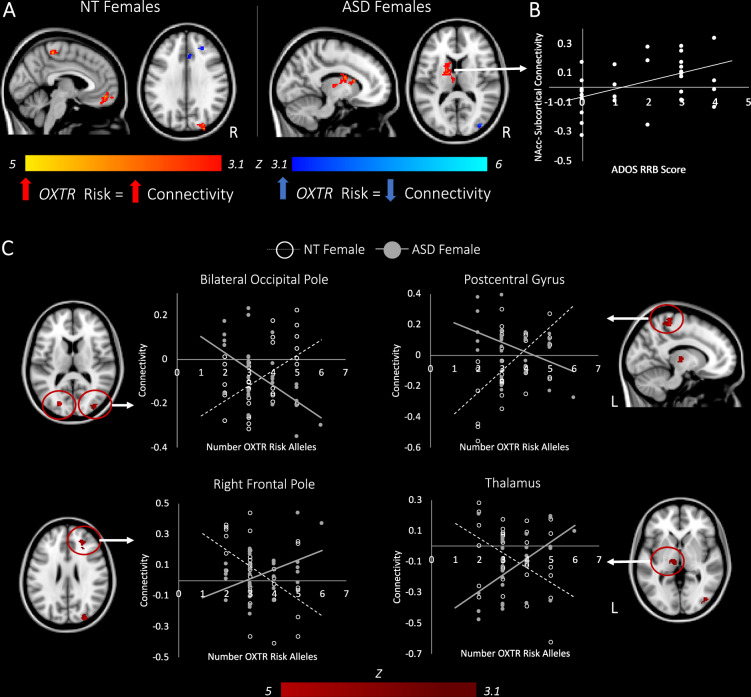
Effects of *OXTR* genetic risk on connectivity of the NAcc in females with and without ASD. **a** Brain regions showing greater connectivity with the NAcc as a function of increased *OXTR* genetic risk are shown in red/yellow. Areas showing reduced functional connectivity with the NAcc as a function of increased *OXTR* genetic risk are shown in blue/cyan. **b** In females with ASD, greater NAcc-Subcortical connectivity is associated with higher scores on the restricted and repetitive behaviors (RRB) scale of the Autism Diagnostic Observation Scale (ADOS-2)^47^. **c** Areas in which diagnostic group differences were observed in the relationship between *OXTR* risk-allele dosage and NAcc functional connectivity. Graphs are for illustrative purposes and show the relationship between NAcc connectivity and number of *OXTR* risk alleles for each participant. Maps are shown at *z* > 3.1, corrected for multiple comparisons at *p* < 0.05. NT neurotypical; ASD autism spectrum disorder.

In females with ASD, greater numbers of ASD-associated variants in the *OXTR* gene were associated with stronger connectivity between the NAcc and subcortical brain regions involved in implicit learning and sensory processing including the dorsal striatum (caudate, putamen) and thalamus, as well as weaker connectivity with visual cortex (Fig. 2a; Supplementary Table 3). We conducted exploratory analyses whereby parameter estimates of connectivity were extracted from these clusters and correlated with scores on the two ADOS subscales, as well as the Social Cognition subscale of the SRS. A Spearman rank-order correlation indicated that increased NAcc-subcortical connectivity in ASD females was related to higher RRB scores on the ADOS (*r*_*s*_ = 0.42, *p* = 0.02; Fig. 2b); however, this finding did not survive correction for the number of tests performed (*n* = 6). Higher RRB scores in females with ASD also positively correlated with increased *OXTR* genetic risk (*r*_*s*_ = 0.46, *p* = 0.01); however, NAcc-subcortical connectivity did not mediate the relationship between genetic risk and RRB.

In contrast, in NT females, increased genetic risk for ASD in the *OXTR* was associated with increased connectivity between the NAcc and frontal brain areas implicated in social cognition/mentalizing (i.e., frontal medial cortex, frontal pole), as well as sensory brain regions (pre/postcentral gyrus, occipital pole). As *OXTR* risk-allele dosage increased, NT females also showed reduced functional connectivity between the NAcc, anterior cingulate gyrus, paracingulate, superior frontal gyrus, and frontal pole (Fig. 2a; Supplementary Table 3).

Between-group comparisons revealed that aggregate *OXTR* risk differentially modulated NAcc connectivity in NT and ASD females in sensory brain regions (bilateral occipital cortex, postcentral gyrus, thalamus), as well as a region in the frontal pole implicated in working memory (Fig. 2c; Supplementary Table 3). These between-group differences held in a subsample of NT and ASD females matched for IQ (Supplementary Table 4). To confirm that results were not biased by ancestry, the first two components from multidimensional scaling of genome-wide data were controlled for in a correlation analysis between cumulative risk-allele dosage and parameter estimates extracted from brain regions modulated by *OXTR* risk at the whole-brain level; results in both NT and ASD females remained significant.

### Cumulative genetic risk: sex-specific effects on NAcc connectivity

A significant *OXTR* risk-allele dosage by sex by diagnostic group ANCOVA indicated that the effects of *OXTR* in NT males vs. females differed from those observed in ASD males vs. females (Supplementary Fig. 3). To qualify these findings, simple two-way interactions were tested.

**Fig. 3:**
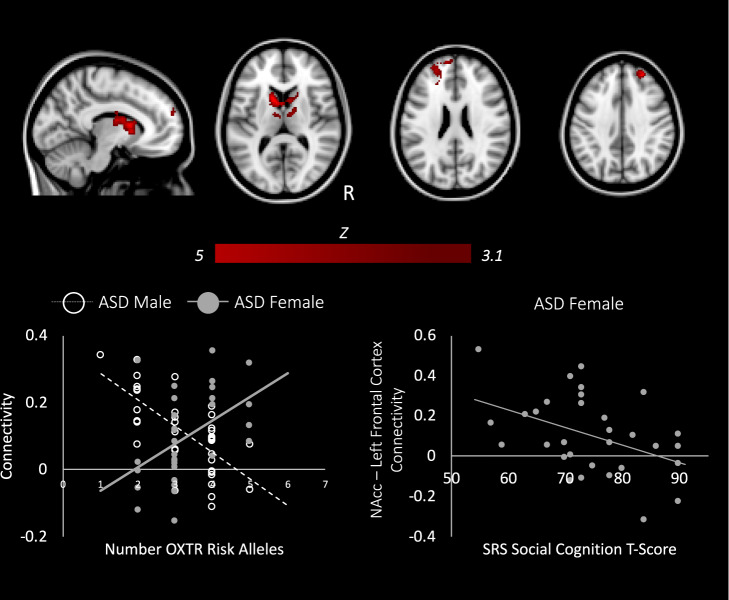
Distinct effects of *OXTR* variants on brain connectivity in males and females with autism. Top: Brain areas showing between-group differences as a function of sex and aggregate *OXTR* risk (z > 3.1, *p* < 0.05). Bottom, right: In ASD females, increased connectivity between the NAcc and left frontal pole is associated with lower social cognition *T-*scores on the SRS (indicative of less severe social-cognitive symptomatology).

Sex significantly modulated the relationship between *OXTR* genetic risk and NAcc connectivity in the ASD group only. Relative to their male counterparts, as genetic risk for ASD increased, females with ASD showed significantly greater connectivity between the NAcc and regions of the mesolimbic reward system, including the caudate, pallidum, and putamen, as well as bilateral thalamus, right prefrontal cortex, and left medial prefrontal cortex (Fig. 3, Supplementary Table 5). This region of the left prefrontal cortex that showed greater connectivity with the NAcc in ASD females with high genetic risk (compared to ASD males with high risk) is the same region where NT males show stronger connectivity (compared to ASD males with high risk) as a function of increasing *OXTR* aggregate risk (Supplementary Fig. 2)^45^. Furthermore, previous work in NT males has shown that increased NAcc-left prefrontal connectivity is associated with better social cognition as measured by the SRS^45^. We thus had an a priori hypothesis that NAcc-left prefrontal connectivity would be related to scores on the SRS social cognition subscale. Indeed, in females with ASD, extracting parameter estimates indexing NAcc-left prefrontal connectivity showed that stronger reward-frontal cortex connectivity was related to better social cognition as measured by the SRS (*r* = −0.43, *p* = 0.02; Fig. 3). While *OXTR* risk allele-dosage was negatively correlated with SRS scores in females with ASD (*r*_*s*_ = −0.4, *p* < 0.05), NAcc-left prefrontal connectivity did not mediate this relationship. Additional exploratory brain-behavior correlations with ADOS subscales and the social cognition subscale of the SRS were examined for clusters showing a sex-specific effect of *OXTR* risk-allele dosage (i.e., right prefrontal cortex and subcortical brain regions shown in Fig. 3); results were not significant.

Additional analyses were conducted to test the extent to which gene-brain-behavior patterns observed in ASD females and NT males were indeed similar. First, at the whole-brain level, aggregate *OXTR* risk differentially modulated NAcc connectivity in NT males vs. ASD females in subcortical but not in prefrontal brain regions (Supplementary Fig. 4). Second, parameter estimates were extracted from the left prefrontal brain areas where ASD females and NT males showed greater connectivity with the NAcc as a function of *OXTR* genetic risk relative to ASD males (i.e., ASD female > ASD male (Fig. 3) and NT male > ASD male (Supplementary Fig. 2)); a two-tailed *t*-test using these connectivity indices revealed no significant differences between ASD females and NT males (*p* = 0.26), indicating that connectivity between the NAcc and the left prefrontal cortex did not vary significantly between the two groups. Third, an analysis of covariance (ANCOVA), controlling for the same variables used in our original bottom-up between-group analyses, was used to test for homogeneity of regression slopes (i.e., the slope of the relationship between *OXTR* risk-allele dosage and NAcc-left prefrontal cortex connectivity) between ASD females and NT males; the Group × *OXTR* comparison was not significant (*β* = −0.06, SE = 0.04, *t* = −1.55, *p* = 0.13), indicating no significant differences in slopes between ASD females and NT males.

## Discussion

Here, we report that common variants in the *OXTR* modulate reward network functional connectivity in both a sex- and diagnosis-dependent manner. Directly comparing males and females with ASD, we found sex-specific associations between *OXTR* variants and brain circuitry such that in the presence of increased genetic risk, females showed greater connectivity between the reward network and prefrontal brain regions important for social cognition and inhibitory control, whereas males showed the opposite pattern. Further, in females with ASD, greater NAcc connectivity with left prefrontal regions was also associated with better functioning on the social cognition subscale of the SRS. Remarkably, these gene-brain-behavior results directly mirror our previously reported findings in NT males, who showed the same pattern whereby NAcc-left prefrontal connectivity increased as a function of increased *OXTR* risk-allele-dosage and was related to better social cognition scores on the SRS^45^. These findings suggest that the relationship between *OXTR* genetic risk and brain connectivity in females with ASD, rather than being more similar to ASD males, are more akin to neurotypical males both in terms of reward network-frontal connectivity and brain-behavior relationships. These findings are in agreement with a female protective model of ASD susceptibility in which ASD risk variants (such as those in the *OXTR*) interact with other genes that are expressed in a sex-differential and regionally-specific manner throughout the brain, resulting in the manifestation of sex-specific neuroendophenotypes in individuals with ASD. Gene expression analyses in post-mortem human brain samples have shown that individuals with ASD (compared to neurotypical individuals) and males (compared to females) show downregulation of genes with neural and synaptic functions, as well as upregulation of genes involved in neuroimmune and inflammatory processes^34,58^. Thus, male-typical gene expression profiles may create a background on which ASD-risk genes are likely to have more penetrant/deleterious effects whereas female gene expression profiles may act to buffer the effects of ASD-risk variants on neural circuitry^34^. Our data support this theory, as we found that cumulative genetic risk for ASD in the *OXTR* has more penetrant effects on connectivity within the reward network in males with ASD compared to NT males^45^, and that females with ASD may be in part buffered from the neurobehavioral effects of high *OXTR* genetic risk through formation of compensatory NAcc-prefrontal connectivity. Interestingly, behavioral research suggests that high functioning females with ASD show greater social motivation compared to their male counterparts^59–61^, a finding which has been attributed to the ability of females with higher IQ to mask their social-communicative deficits^60,62^. Our findings of increased connectivity between brain regions involved in reward processing and prefrontal brain areas important for metalizing in high functioning females with ASD may provide a neurobiological mechanism supporting this behavioral process.

A second feature of ASD females that distinguished them from both ASD males and NT females was that higher *OXTR* risk was related to greater connectivity between the NAcc and subcortical brain regions, a pattern not observed in any other group. Subcortical brain areas in the striatum are known to play a critical role in implicit learning of motor repertoires through cortico-basal ganglia feedback circuits^63,64^, and indeed this increased NAcc-subcortical connectivity was associated with higher restricted interests and repetitive behavior (RRB) scores on the ADOS in females with ASD. Notably, a sex-bias in expression of repetitive behaviors has been documented in individuals with ASD whereby males generally display more repetitive behaviors and restricted interests relative to females^65^. Our imaging-genetics results in ASD females suggest that increased genetic risk in the *OXTR*, as well as associated up-regulation of connectivity between the reward network and subcortical brain regions, may put ASD females at a greater likelihood of expressing RRBs. Although females in our study did not display more severe RRB than males with ASD, we also did not see the typical pattern whereby males with ASD show higher RRB relative to females.

That the *OXTR* risk variants investigated in this study would affect frontal and subcortical brain regions in a sex-specific manner are in line with findings from studies using intranasal oxytocin to alter neural activity. In adult neurotypical females, administration of intranasal oxytocin increases connectivity between subcortical brain regions involved in reward and social-emotional processing, and, importantly the effects of oxytocin are greatest in women with higher autistic traits^39^. Sex differences in the effects of intranasal oxytocin have been documented in neurotypical adults such that oxytocin increases brain activity in the caudate and putamen in men, while decreasing activity in these brain regions in women^66^. Furthermore, these sex-dependent effects of oxytocin on the brain interact with genetic variants in the *OXTR*. For instance, for the rs53576 SNP on which the A allele has been associated with increased rates of ASD diagnosis, male GG homozygotes (i.e., the non-risk group) show greater caudate activity during a game eliciting social cooperation under oxytocin vs. placebo, while female GG carriers have greater caudate activity in the placebo condition^67^. Similarly, for the rs1042778 SNP for which the G allele confers ASD-risk, male TT homozygotes (i.e., the non-risk group) display greater increases in amygdala activity in response to angry faces compared to risk-allele carriers, while no significant difference is observed between female risk and non-risk groups^68^. How these results may translate to males and females with ASD and how the modulatory effects of oxytocin may be affected by cumulative genetic risk for ASD in the *OXTR* have yet to be empirically determined. Taken together with previous data, our findings suggest that in addition to investigating the effect of sex on oxytocin treatment outcomes, it is critical that future work also consider sex x diagnosis interactions, as ASD males and females are likely to show different neural responses, at baseline and in response to treatment, from their NT male and female counterparts. Notably, recent research suggests that the effects of oxytocin on neural activity may be modulated not only by sex, but also by age^20^, pointing to the need to study the effects of pharmacological treatments in the context of sex-specific neurodevelopmental trajectories.

In NT females, greater genetic risk for ASD in the *OXTR* was related to reduced functional connectivity between the NAcc and anterior cingulate (ACC), and increased connectivity with medial orbital frontal cortex (OFC). Both the ACC and OFC are components of the limbic fronto-striatal circuit, which plays a role in reward processing and goal directed planning based on distant outcomes^64^. Neuroanatomically, when a rewarding stimulus is given (i.e., a smiling face, money, etc.), dopaminergic projections from the midbrain encode reward prediction error^69^ and signal to the ventral striatum and frontal cortex, which encode the subjective reward value of the stimulus^70^. Thus, the ventral striatum and frontal cortex form a feedback loop that is critical for implicit learning of the relationship between reward prediction and likely outcomes. Our findings in NT females suggest that variability in *OXTR* genotypes impact the relative balance of connectivity between nodes of the fronto-striatal loop in NT females, possibly affecting signaling from the frontal lobe back to subcortical brain regions to impact decision making based on previous reward. Notably, there are sex- and age-dependent differences in structural connectivity between the NAcc and OFC in neurotypical individuals with males showing earlier peak in structural connectivity between these two regions relative to females^71^. The developmental trajectory of accumbo-frontal connectivity remains to be explored in males and females with ASD and is an important future direction.

In sum, this study examined resting-state functional connectivity of the nucleus accumbens in females with and without ASD to investigate how ASD risk-allele dosage in the *OXTR* relates to between-subject heterogeneity in network connectivity. Critically, we investigated sex differences in the effects of genetic risk on the brain by comparing cohorts of males and females with ASD. Taken together, our results confirm previous evidence that *OXTR* risk variants are indeed associated with distinct neuroendophenotypes in both NT and ASD individuals. Our findings show that the specific brain regions affected and the direction of the observed effect varies as a function of both sex and ASD-diagnostic status. These findings have important implications for treatment studies investigating the effects of intranasal oxytocin on brain activity as they suggest that males and females may have different responses to pharmacological treatment depending on their genetic background and diagnostic status. More broadly, these findings underscore the importance of including females in studies investigating the neural basis of ASD, which in addition to providing insights into the etiology of ASD, can inform our understanding of how variability at the genetic, neural, and behavioral level gives rise to sex differences in rates of ASD diagnosis, and ultimately inform personalized approaches to the treatment of ASD.

## Supplementary information

Supplemental Material
