# Supplementary material for: Imaging-genetics of sex differences in ASD: distinct effects of OXTR variants on brain connectivity
Source: Transl Psychiatry. 2020 Mar 3;10:82. doi: 10.1038/s41398-020-0750-9 (PMC7054353; doi:10.1038/s41398-020-0750-9)
Supplement: Supplementary file 1 — Supplemental Material [file 41398_2020_750_MOESM1_ESM.docx]

**SUPPLEMENTARY MATERIALS**

**Supplementary Figure 1.** Nucleus accumbens (NAcc) whole-brain connectivity in males with and without ASD. Red/yellow indicate positive connectivity with the NAcc seed; blue/cyan indicate negative connectivity with the NAcc seed. Maps are shown at z>3.1, corrected for multiple comparisons at p<0.05. NT, neurotypical; ASD, autism spectrum disorder.

**Supplementary Figure 2.** Effects of *OXTR* genetic risk on connectivity of the NAcc in males with and without ASD. Brain regions showing greater connectivity with the NAcc as a function of increased *OXTR* genetic risk are shown in red/yellow. Areas showing reduced functional connectivity with the NAcc as a function of increased *OXTR* genetic risk are shown in blue/cyan. Areas in which *OXTR* risk-allele dosage differentially modulates NAcc connectivity in NT males vs. ASD males are shown in maroon. Maps are shown at z>3.1, corrected for multiple comparisons at p<0.05. NT, neurotypical; ASD, autism spectrum disorder.

**Supplementary Figure 3.** Group by sex by *OXTR* interactions showing brain regions where the effects of *OXTR* in NT males vs. females differed from those observed in ASD males vs. females. Graphs are for illustrative purposes and show the relationship between NAcc connectivity and number of *OXTR* risk alleles for each participant. Maps are shown at z>3.1, corrected for multiple comparisons at p<0.05. NT, neurotypical; ASD, autism spectrum disorder.


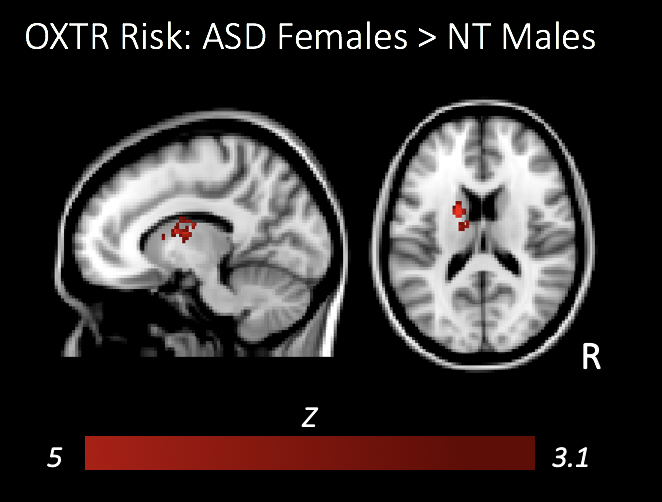


**Supplementary Figure 4.** *OXTR* risk-allele dosage differentially modulated NAcc connectivity in ASD females vs. NT males. There were no regions where NT males showed greater connectivity with the NAcc as a function of increasing *OXTR* risk-allele dosage relative to ASD females. Maps are shown at z>3.1, corrected for multiple comparisons at p<0.05. NT, neurotypical; ASD, autism spectrum disorder.

**Supplementary Table 1.** Medication Usage. Fifteen ASD females were medication free (46.9%), 9 were taking one psychotropic medication (28.1%), and 8 were taking two or more medications (25%). Nineteen ASD males were medication free (51.4%), 11 were taking one psychotropic medication (29.7%), and 7 were taking two or more medications (18.9%). All NT participants were medication free.

**Supplementary Table 2.** Descriptive information for ASD-Associated *OXTR* variants. Odds ratios (Di Napoli et al., 2014; LoParo & Waldman, 2015). eQTL evidence reported from the Genotype-Tissue Expression Project (GTEx; Lonsdale et al., 2008). eQTL; expression quantitate trait loci.

**Supplementary Table 3.** Peak coordinates of brain regions modulated by variability in *OXTR* risk-allele dosage; z>3.1, corrected for multiple comparisons at p<0.05.

**Supplementary Table 4.** Results in a subset of IQ-matched females with and without ASD. Follow-up analyses were performed by removing 5 NT females with the highest IQ scores, and 5 ASD females with the lowest IQ scores, thereby creating two female groups (N_ASD_=27, N_TD_=18) matched for verbal IQ (M_ASD_=105.4, M_TD_=108.3, p=0.48), nonverbal IQ (M_ASD_=102.1, M_TD_=107.0, p=0.24), and full-scale IQ (M_ASD_=103.1, M_TD_=109.1, p=0.14). In this smaller group of participants, parameter estimates were extracted from brain regions showing a main effect of *OXTR* risk-allele dosage on NAcc connectivity in the larger sample (i.e., regions displayed in Figure 2A,B), as well as brain regions where an interaction was detected (i.e., regions displayed in Figure 2C); parameter estimates were then correlated with *OXTR* risk-allele dosage. All reported results held in this smaller sample of NT and ASD females matched for IQ. Correlation coefficients, as well as two-tailed p-values are presented for each of the main clusters.

**Supplementary Table 5.** Peak coordinates of sex differences in NAcc functional connectivity as a function of *OXTR* risk in youth with ASD; z>3.1, corrected for multiple comparisons at p<0.05.
